# Spexin Regulates Hypothalamic Leptin Action on Feeding Behavior

**DOI:** 10.3390/biom12020236

**Published:** 2022-01-31

**Authors:** Bora Jeong, Kwang-Kon Kim, Tae-Hwan Lee, Han-Rae Kim, Byong-Seo Park, Jeong-Woo Park, Jin-Kwon Jeong, Jae-Young Seong, Byung-Ju Lee

**Affiliations:** 1 Department of Biological Sciences, College of Natural Sciences, University of Ulsan, Ulsan 44610, Korea; boraring@naver.com (B.J.); fromfriend77@gmail.com (K.-K.K.); saint1033@naver.com (T.-H.L.); bbs0808@naver.com (B.-S.P.); jwpark@ulsan.ac.kr (J.-W.P.); 2 Department of Pharmacology and Physiology, School of Medicine & Health Sciences, The George Washington University, Washington, DC 22037, USA; hrkim@gwu.edu (H.-R.K.); jinkwon0911@gwu.edu (J.-K.J.); 3 Graduate School of Medicine, Korea University, Seoul 02841, Korea

**Keywords:** energy homeostasis, appetite regulation, hypothalamus, spexin

## Abstract

Spexin (SPX) is a recently identified neuropeptide that is believed to play an important role in the regulation of energy homeostasis. Here, we describe a mediating function of SPX in hypothalamic leptin action. Intracerebroventricular (icv) SPX administration induced a decrease in food intake and body weight gain. SPX was found to be expressed in cells expressing leptin receptor ObRb in the mouse hypothalamus. In line with this finding, icv leptin injection increased SPX mRNA in the ObRb-positive cells of the hypothalamus, which was blocked by treatment with a STAT3 inhibitor. Leptin also increased STAT3 binding to the SPX promoter, as measured by chromatin immunoprecipitation assays. In vivo blockade of hypothalamic SPX biosynthesis with an antisense oligodeoxynucleotide (AS ODN) resulted in a diminished leptin effect on food intake and body weight. AS ODN reversed leptin’s effect on the proopiomelanocortin (POMC) mRNA expression and, moreover, decreased leptin-induced STAT3 binding to the POMC promoter sequence. These results suggest that SPX is involved in leptin’s action on POMC gene expression in the hypothalamus and impacts the anorexigenic effects of leptin.

## 1. Introduction

Spexin (SPX), also known as neuropeptide Q, has recently been identified by bioinformatics techniques [1,2]. SPX is a secreted 14-amino-acid peptide that is highly conserved from fish to mammals [3,4,5]. It is widely expressed in the central nervous system and peripheral tissues such as the liver, gonad and kidney in rodents, fish and humans [1,2,3,4,5,6,7]. Accordingly, SPX has been reported to have a variety of functions, including roles in stomach contraction, adrenocortical cell proliferation, cardiovascular and renal function, nociception, feeding and reproduction [1,4,8,9,10,11].

Recent studies have highlighted SPX functions in the control of obesity and energy metabolism based on the observed relationships between SPX and obesity: the circulating level of SPX is low in obese individuals compared to their normal counterparts [11,12,13,14]. In goldfish, intraperitoneal injection of SPX induced anorexia, accompanied by an increase in hypothalamic expression of anorexigenic neuropeptide proopiomelanocortin (POMC), with a simultaneous decrease in orexigenic neuropeptide Y (NPY) and agouti-related peptide (AgRP) [4]. Additional studies using fish and rodents have further attributed the anorexigenic action of SPX to changes in the hypothalamic feeding circuits. Subcutaneous SPX injections in rats decreased feeding and induced weight loss [11]. SPX-knockout in zebrafish resulted in increased food intake together with increased AgRP mRNA [15]. A recent study reported that intracerebroventricular (icv) injection of SPX increased hypothalamic mRNA levels of leptin receptor and melanocortin 4 receptor in mice [16], suggesting a possible role of SPX in the action of leptin in the hypothalamic center for energy homeostasis. However, no evidence is available about detailed SPX interaction with central leptin effects. Therefore, we sought to identify whether SPX is involved in the anorexigenic action of leptin in the hypothalamus.

As an adiposity hormone, leptin is a key regulator for energy metabolism via regulation of the hypothalamic center for energy homeostasis [17,18,19,20]. Leptin receptor, also known as obesity receptor (ObR), especially the long isoform of the ObR (ObRb), is dominantly expressed in the hypothalamus and is essential for leptin signal transduction [21,22,23,24]. Binding of leptin to ObRb results in phosphorylation of signal transducer and activator of transcription 3 (STAT3) via Janus kinase-2 (JAK2) activation [25]. In turn, phosphorylated STAT3 translocates into the nucleus and regulates target gene transcription [26,27]. Within the hypothalamus, leptin regulates food intake and energy expenditure via the arcuate nucleus (ARC), which is home to two opposite-acting neuronal populations, orexigenic AgRP/NPY neurons and anorexigenic POMC neurons [18,19,20,28,29]. Leptin-induced phosphorylation of STAT3 directly regulates POMC and AgRP gene expression in the hypothalamus [30,31,32].

In this study, we found that leptin stimulated hypothalamic SPX expression via STAT3 signaling. Inhibition of hypothalamic SPX synthesis resulted in inhibition of leptin-induced decreases in food intake and body weight gain and a concurrent inhibition of leptin-induced increases in POMC mRNA expression. Therefore, our results provide insights into the previously unidentified function of SPX in the regulation of energy balance.

## 2. Materials and Methods

### 2.1. Animals

Eight-week-old male C57BL/6 mice (Hyochang Science, Daegu, Korea) were used for this study in accordance with the Regulations of the University of Ulsan for the Care and Use of Laboratory Animals (BJL-20-020). To generate transgenic mice, we crossbred *ObRb*-Cre mice (stock number: 008320) with *Ai14* reporter mice (stock number: 007914) and *Rpl22*^HA^ mice (stock number: 011029) purchased from Jackson Laboratory (Bar Harbor, ME). All animals were housed in a humidity- and temperature-controlled room with a 12-h light/dark cycle (lights on from 7:00 to 19:00) with pelleted mouse chow and water *ad libitum*.

### 2.2. Intracerebroventricular (icv) Cannulation and Material Administration

The cannula was introduced into the right lateral ventricle 0.3 mm posterior, 1.0 mm lateral and 2.4 mm ventral to the bregma and secured to the skull with dental cement. After a week recovery period in individual cages, mice were icv-administered experimental materials through the cannula.

For administration of leptin (2.5 μg/2.5 μL, R&D Systems, Minneapolis, MN, USA), mice were fasted for 24 h and slowly injected using a Hamilton syringe (Hamilton, NV, USA) and killed 1~3 h after injection for the determination of molecular changes in POMC and AgRP mRNA. Food intake and body weight gain were measured for 24 h after leptin injection. SPX (NWTPQAMLYLKGAQ, peptide purity 97.7%) was synthesized by Lugen Sci Inc. (Bucheon-si, Korea) and the injected dose was 10 μg/2 μL. S3I-201 (an inhibitor of STAT3 activation, 100 μM, Sigma-Aldrich, St. Louis, MO, USA) was injected 3 h before administration of leptin. M871 (an antagonist for GALR2, 5 nM, Abcam, Cambridge, UK) and SNAP37889 (SNAP, an antagonist for GALR3, 5 nM, Key Organics, Camelford, UK) were injected 1 h prior to leptin administration.

One hour after leptin administration, mice were killed and brain samples were rapidly obtained using the micropunch technique. Part of the caudal diencephalon was coronal sectioned to obtain the ARC, paraventricular nucleus (PVN), lateral hypothalamus (LH) and suprachiasmatic nucleus (SCN) and frozen. Referring to the mouse brain atlas [33], the nuclei were punched out using a micropunching set (Stoelting, Wood Dale, IL, USA).

### 2.3. Measurement of Body Temperature and Locomotor Activity

Eight-week-old male C57BL/6 mice were used for measurement of body temperature and locomotor activity using biotelemetry transmitters (E-mitter, STARR Life Science Corp., Oakmont, PA, USA) as previously described [34]. Briefly, mice were anesthetized with tribromoethanol (250 mg/kg, Sigma-Aldrich) and a biotelemetry transmitter was implanted into the abdominal cavity. Output was monitored by a receiver (ER-4000, STARR Life Science Corp.) laid under each cage. A data acquisition system (Vital View, STARR Life Science Corp.) was used for automatic data collection and analysis. Mice were adapted to the vital view system for 24 h prior to SPX injection.

### 2.4. Antisense Oligodeoxynucleotide Administration

To inhibit endogenous SPX expression in the mouse hypothalamus, antisense (AS) SPX oligodeoxynucleotide (ODN) was designed based on NCBI GenBank^TM^ database (accession number NM_001242345) and synthesized (Lugen Sci Inc.). AS SPX ODN (5′-UGG GCC CCT TCA TGT CCG A-3′) or scrambled (SCR) ODN (5′-AAC CGG TTA CGT CCG TCC GTA ACC-3′) was icv-injected into the lateral ventricle of mice once a day for 2 days and then mice were fasted for 24 h before leptin administration.

### 2.5. Ribo-Tag System

Transgenic mice were generated by crossbreeding *ObRb*-Cre mice and *Rpl22*^HA^ mice for the Ribo-Tag system (*ObRb*-Cre;*Rpl22*^HA^ mice). Because the *Rpl22*^HA^ mice have a *loxP*-flanked wild-type exon 4 and a modified exon 4 tagged with hemagglutinin (HA), wild-type exon 4 is replaced by the *Rpl22*^HA^ exon 4 after crossing the Cre recombinase-expressing mice [35]. RNA isolation with the Ribo-Tag system was conducted as previously described [35,36]. Briefly, dissected hypothalamus samples were collected from animals and homogenized. RNA was extracted from 10% of the cleared lysate and used as input. The remaining lysate was incubated with mouse anti-HA antibody for 4 h at 4 °C followed by the addition of protein G agarose beads (LGP-1018B, Lugen Sci Inc.) and overnight incubation at 4 °C. The beads were washed three times in high-salt solution. The bound ribosomes and RNA were separated from the beads with 30 sec of vortexing, and RNA was further purified using a QIAGEN RNeasy Micro Kit (74034, Qiagen, Hilden, Germany). After RNA isolation, we obtained 10–20 ng of RNA sample/hypothalamus. The RNA samples were then subjected to qRT-PCR analysis.

### 2.6. Quantitative Real-Time PCR

Total RNA was isolated from the mediobasal hypothalamus (MBH) or micropunched hypothalamic ARC using Sensi-TriJol reagent (Lugen Sci Inc.) and reverse transcribed with MMLV reverse transcriptase (Beams Biotechnology, Seongnam-si, Korea). The resulting cDNA samples were amplified by real-time PCR with the following primer sets: SPX forward primer, 5′-CTG GTG CTG TCT GCG CTG-3′; SPX reverse primer, 5′-CTG GGT TTC GTC TTT CTG G-3′; ObRb forward primer, 5′-ACC ACA ACT TTC ATT CTC GGG-3′; ObRb reverse primer, 5′-CTC ACC AGT CAA AAG CAC AC-3′; POMC forward primer, 5′-GCT AGG TAA CAA ACG AAT GG-3′; POMC reverse primer, 5′-GCA TTT TCT GTG CTT TCT CT-3′; AgRP forward primer, 5′AAT GTT GCT GAG TTG TGT TCT G-3′; AgRP reverse primer, 5′-GGC CAT TCA GAC TTA GAC CTG-3; β-actin forward primer, 5′-TGG AAT CCT GTG GCA TCC ATG AAA C-3′, β-actin reverse primer, 5′-TAA AAC GCA GCT CAG TAA CAG TCC G-3′. Quantitative real-time PCR (qRT-PCR) was reacted using an Evagreen PCR Mastermix (Applied Biological Materials Inc., Richmond, BC, Canada). Relative mRNA levels were normalized to β-actin level and calculated using the 2^−ΔΔCt^ method [37].

### 2.7. Immunohistochemistry

Mice were transcardially perfused with phosphate buffer (PB, 0.1 M, pH 7.4) and fixed in fresh 4% paraformaldehyde. Isolated brains were post-fixed in 4% paraformaldehyde and cut to obtain coronal brain sections (50 μm in thickness) using a vibratome (Leica Microsystems, Wetzlar, Germany). Sections including the hypothalamic ARC were selected using a stereo microscope (Carl Zeiss Microimaging Inc., Thornwood, NY, USA) and incubated with 3% hydrogen peroxide (H_2_O_2_) in 0.1 M phosphate buffer (PB, pH 7.4) for 10 min to block endogenous peroxidase activity. The sections were incubated with 3% normal goat serum (Vector Laboratories, Burlingame, CA, USA) in 0.3% Triton X-100 (Sigma-Aldrich, St. Louis, MO, USA) for 1 h. After washing with PB, sections were treated with primary antibodies as follows: rabbit anti-SPX antibody (1:500, Phoenix Pharmaceuticals, Inc., Burlingame, CA, USA) and mouse anti-HA antibody (1:1000, BioLegend, San Diego, CA, USA) overnight at 4 °C. The next day, samples were washed with PB and incubated with goat anti-rabbit Alexa Fluor 488 (1:1000, Invitrogen, Gaithersburg, MD, USA) or goat anti-mouse Alexa Fluor 488 (1:1000, Invitrogen) antibody for 3 h at room temperature for the immunofluorescence. The sections were mounted onto slides and covered with coverslips. The images were obtained by using FV1200 confocal microscope (Olympus, Tokyo, Japan).

### 2.8. Cell Culture and Luciferase Assay

mHypoA cells (CELLutions Biosystems Inc., Burlington, ON, Canada) were cultured in high glucose Dulbecco’s modified Eagle medium (4.5 g/L) supplemented with 10% fetal bovine serum and streptomycin (0.1 mg/mL)-penicillin (100 U/mL) at 37 °C with an atmosphere with 5% CO_2_. Cells were transfected with 300 ng of pGL3 luciferase reporter plasmid containing POMC promoter (pGL3-POMC) [38] and 30 ng of a β-galactosidase reporter plasmid (pCMV-β-gal; Clontech, Palo Alto, CA, USA) per well using jetPRIME^®^ transfection reagent (Polyplus-transfection^®^ SA, Illkirch, France). Indicated test materials were treated and luciferase assay was performed 24 h after transfection using luciferase reporter assay system (Promega, Madison, WI, USA) according to the manufacturer’s protocols. Transfection efficiency was normalized by β-galactosidase assays.

### 2.9. Chromatin Immunoprecipitation Assays

Mice MBHs were separated and chromatin immunoprecipitation (ChIP) assays were performed using ChIP Assay kits (Millipore, Billerica, MA, USA) according to the manufacturer’s protocol. Briefly, diluted chromatin samples reacted with 5 μg anti-STAT3 antibody (Cell signaling Technology, Beverly, MA, USA) and protein G magnetic beads at 4 °C with rotation for overnight. The magnetic beads were eliminated using a magnetic separator and DNA was purified by spin columns from protein-DNA complex. We found a STAT3 binding site at the 5′-flanking region of mouse SPX, POMC and AgRP. Based on this analysis, PCR amplification was performed using the following primer sets; SPX at -1235, forward, 5′-GTG TGA AGT TAG AGG ACA AT-3′; SPX at -1235 reverse, 5′-GAC AGG CAA GTA GAA ACA TA-3′; POMC at -3254 forward, 5′-CCT CTG TCC AGT TCT AAG-3′; POMC at -3254 reverse, 5′-CGC TCT TCT CTC TTC TTT-3′; AgRP at -2319 forward, 5′-CAG GAA CCT TAG GTA GAA-3′; AgRP at -2319 reverse, 5′-GGC CCT CTG ATC TTA AT-3′.

### 2.10. Statistics

Data were analyzed with Student’s *t*-test to compare between two groups using GraphPad Prism 6.0 software (GraphPad Software, San Diego, CA, USA). All data are presented as mean ± SEM.

## 3. Results

### 3.1. Central SPX Induces Anorexia in Mice

**Figure 1:**
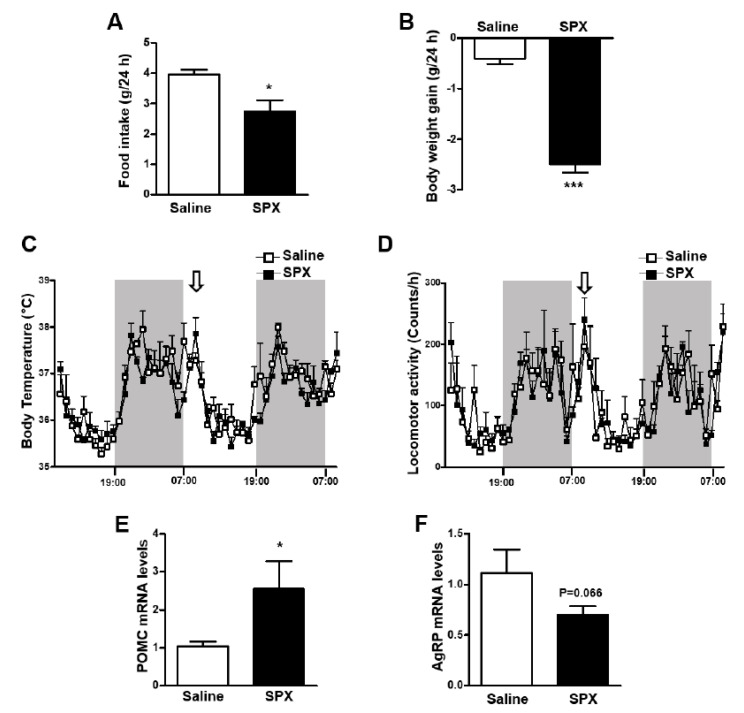
**Effects of icv SPX administration on food intake, body weight and POMC and AgRP mRNA levels.** Eight-week-old male mice were icv-injected with saline or SPX (10 μg). Food intake (**A**) and body weight gain (**B**) were measured for 24 h after injection. Body temperature (**C**) and locomotor activity (**D**) were observed for 48 h using a vital-view telemetry system. SPX was injected at the beginning of light period as indicated with arrows. Shaded areas represent the dark period. No difference was observed between injection with saline and SPX (n = 4/group). POMC (**E**) and AgRP (**F**) mRNA levels were assessed by qRT-PCR analysis. Data are presented as mean ± S.E.M. *, *p* < 0.05; ***, *p* < 0.001.

Previous studies reported that SPX plays a role in the regulation of feeding and energy metabolism [11,15,16,39]. In particular, central administration of SPX into the mouse brain resulted in a significant reduction in food consumption [16]. Here, we also found that icv administration of SPX reduced food intake and body weight gain compared with saline-injected control mice (Figure 1A,B). However, there was no difference in body temperature or locomotor activity between experimental groups (Figure 1C,D). Because SPX induced a decrease in food intake, we next investigated the SPX-induced change in POMC and AgRP mRNA levels. POMC mRNA expression was significantly increased in the mouse MBH after icv administration of SPX, whereas AgRP mRNA levels were not changed significantly (Figure 1E,F). These results indicate that the anorexigenic action of central SPX can mainly be attributed to alterations in POMC expression.

### 3.2. Leptin Increases SPX Expression via STAT3 Activation in the Mouse Hypothalamus

**Figure 2:**
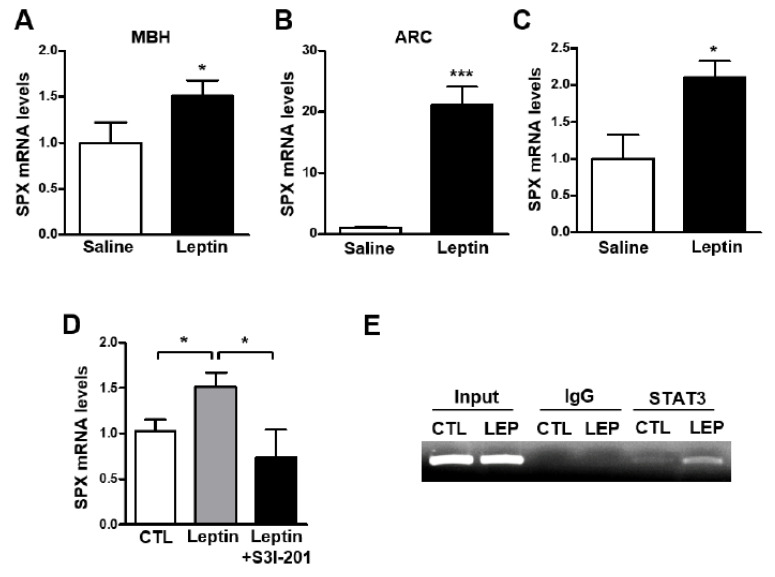
**Effect of leptin on SPX expression via STAT3 activation.** To determine the effect of leptin on SPX expression, eight-week-old male mice were fasted for 24 h and icv-injected with leptin (2.5 μg); tissue samples were collected 1 h after leptin injection. (**A**,**B**) Total RNA was isolated from the mediobasal hypothalamus (MBH) (**A**) and arcuate nucleus (ARC) (**B**) and analyzed with qRT-PCR. (**C**) Leptin was icv-injected into *ObRb*-Cre;*Rpl22*^HA^ mice to analyze leptin-induced SPX mRNA level in ObRb-expressing cells. SPX mRNA was determined using qRT-PCR of MBH RNA samples immunoprecipitated with HA antibody. (**D**) Mice were icv-injected with 100 μM of S3I-201, an inhibitor of STAT3 activation, and then after 3 h, icv-injected with leptin. After 1 h, MBH SPX mRNA was analyzed by qRT-PCR. (**E**) ChIP assays were performed to verify whether STAT3 directly binds to the SPX promoter and whether leptin affects this binding. Mice were icv-injected with either saline (CTL) or leptin (LEP) and nuclear DNA samples from their MBH were immunoprecipitated with anti-STAT3 antibody or mouse IgG. Immunoprecipitates were analyzed by PCR amplification using a primer set specific to the mouse SPX promoter. Input represents the DNA extracted from the mouse MBH before immunoprecipitation. Data are presented as mean ± S.E.M. *, *p* < 0.05; ***, *p* < 0.001.

In a recent study, icv SPX injection increased leptin receptor ObRb mRNA levels in the mouse hypothalamus [16], suggesting a role for SPX in the action of leptin in the hypothalamus. Thus, to identify the relationship between leptin and SPX in the mouse hypothalamus, we analyzed SPX mRNA levels after icv administration of leptin. Interestingly, leptin administration resulted in markedly increased SPX mRNA levels in the MBH and ARC (Figure 2A,B), but not in other hypothalamic regions including the PVN, LH and SCN (Supplementary Figure S1). To further confirm the specific regulation of SPX expression by leptin, we examined the immunoreactivity of SPX in cells expressing ObRb in the mouse hypothalamus using *ObRb-Cre;Ai14* reporter mice (Supplementary Figure S2), which specifically express tdTomato signals in ObRb-positive cells. Many immuno-positive SPX signals were found in tdTomato-expressing cells in the ARC. Next, to determine the effect of leptin on SPX expression specifically in ObRb-positive cells, we used a Ribo-Tag system of transgenic *ObRb-Cre;Rpl22^HA:^Ai14* mice that express HA-tagged ribosomal protein Rpl22 and tdTomato signals in ObRb-expressing cells (Supplementary Figure S3). Using these transgenic mice, we found that icv leptin administration increased SPX mRNA translating in hypothalamic ObRb-positive cells (Figure 2C). These findings indicate that SPX is regulated by leptin action presumably through the hypothalamic ARC.

Therefore, we next investigated whether activation of STAT3 signaling is essential for leptin-induced SPX expression. Mice were icv-injected with leptin (2.5 μg) with or without S3I-201, an inhibitor of STAT3 activation. Leptin’s effect on the increase in SPX mRNA level was blocked by S3I-201 in the MBH (Figure 2D). Moreover, ChIP assays revealed STAT3 binding to its binding site on the SPX gene promoter, which was potentiated by leptin (Figure 2E). Together these results suggest that leptin stimulates SPX expression via STAT3 signaling in hypothalamic cells expressing ObRb.

### 3.3. SPX Inhibition Blocks Leptin’s Anorexigenic Action

**Figure 3:**
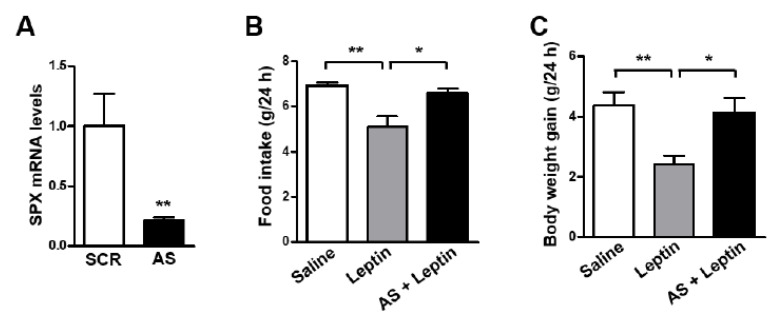
**Effect of SPX synthesis blockade on the anorexigenic effects of leptin.** To inhibit SPX synthesis in the brain, eight-week-old male mice were icv-injected with 2 μg of antisense (AS) or scrambled (SCR) oligodeoxynucleotide (ODN) once a day for 2 days. (**A**) MBH samples were collected at 24 h after the second ODN injection and SPX mRNA levels were determined using qRT-PCR. (**B**,**C**) Mice injected twice with AS ODN were fasted for 24 h before icv leptin (2.5 μg) injection. Food intake (**B**) and body weight gain (**C**) were measured for 24 h after leptin injection. Data are presented as mean ± S.E.M. *, *p* < 0.05; **, *p* < 0.01.

Our results revealed that SPX induces anorexia and leptin increases SPX expression via STAT3 signaling in the mouse hypothalamus, suggesting a possible role for SPX in the mediation of leptin’s anorexigenic effects in the hypothalamus. To examine this possibility, we blocked hypothalamic SPX synthesis by icv injection of SPX AS ODN before icv leptin administration and measured animals’ food intake and body weight. AS ODN markedly decreased SPX mRNA level in the MBH (Figure 3A). Interestingly, leptin-induced decreases in food intake (Figure 3B) and body weight gain (Figure 3C) were significantly reversed by AS ODN treatment.

**Figure 4:**
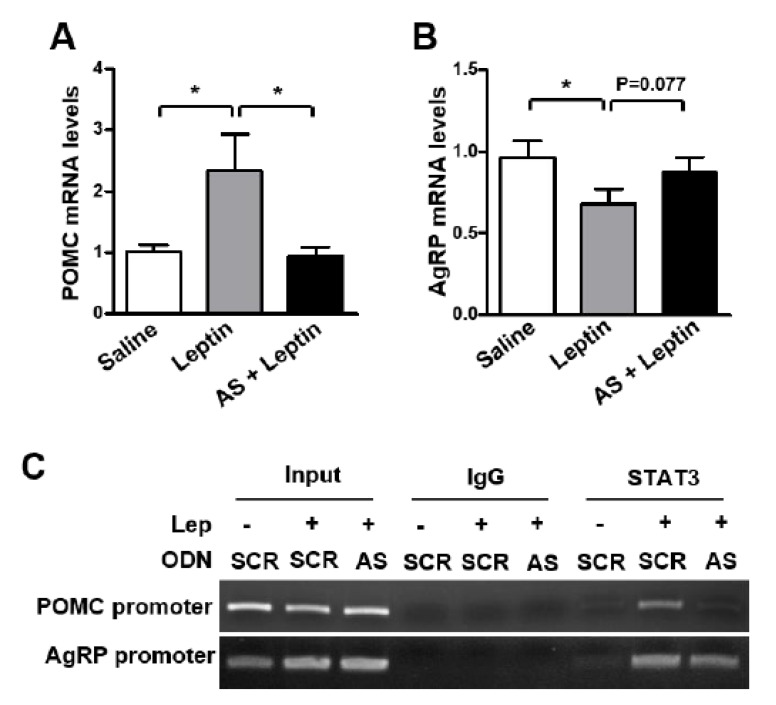
**Effect of SPX synthesis blockade on leptin-induced changes in POMC and AgRP expression.** Eight-week-old male mice were icv-injected with 2 μg of the AS or SCR ODN once a day for 2 days and fasted for 24 h after the second ODN injection. Then, mice were icv-injected with leptin (2.5 μg) and MBH samples were collected 3 h after leptin injection. (**A**,**B**) RNA samples were isolated from MBH tissues and were analyzed to determine POMC (**A**) and AgRP (**B**) mRNA levels using qRT-PCR. (**C**) ChIP assays showing the effect of AS ODN-mediated SPX synthesis blockade on the leptin (LEP)-induced increase in STAT3 binding onto the POMC and AgRP promoters. Nuclear DNA samples from MBH tissues were immunoprecipitated with anti-STAT3 antibody or mouse IgG and were analyzed by PCR amplification using primers specific to mouse POMC and AgRP promoters. Input represents the DNA extracted from the MBH before immunoprecipitation. Data are presented as mean ± S.E.M. *, *p* < 0.05.

Moreover, the leptin-induced increase in POMC mRNA level was attenuated by AS ODN (Figure 4A), whereas no significant effect of AS ODN was observed on the AgRP mRNA level (Figure 4B). Surprisingly, AS ODN administration markedly diminished leptin-induced STAT3 binding onto POMC and AgRP promoters in the mouse hypothalamus (Figure 4C). These results indicate that SPX plays an important role in the effect of leptin on anorexia and POMC expression.

### 3.4. GALR2 Is Important in the Anorexigenic Action of Leptin-Induced SPX

**Figure 5:**
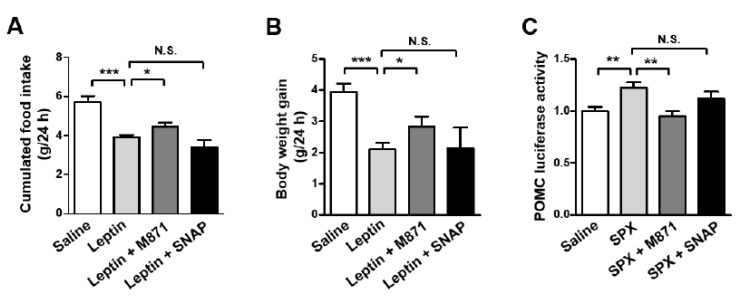
**Anorexigenic effect of leptin-induced SPX via GALR2.** (**A**,**B**) Eight-week-old male mice were fasted for 24 h and icv-injected with M871 (GALR2 antagonist, 5 nM) or SNAP (GALR3 antagonist, 5 nM) 1 h before icv leptin administration. Food intake (**A**) and body weight gain (**B**) were measured for 24 h after leptin injection. (**C**) Luciferase reporter constructs containing the 5′-flanking region of POMC (300 ng) were transfected into mHypoA cells. After 24 h, cells were pre-treated with M871 (1 μM) or SNAP (1 μM) at 30 min before SPX (300 ng/mL) treatment. Then, cells were harvested for luciferase and β-galactosidase assays at 1 h after SPX treatment. Data are presented as mean ± S.E.M. *, *p* < 0.05; **, *p* < 0.01; ***, *p* < 0.001.

Previously, we reported galanin receptor 2 (GALR2) and GALR3 as receptors for SPX as well as galanin [6]. Thus, we sought to identify whether SPX action on leptin-induced anorexia is mediated via GALR2 and/or GALR3. Mice were icv-administered with M871 (GALR2 antagonist) or SNAP (GALR3 antagonist) 1 h before icv leptin injection and their food intake and body weight gain were measured over 24 h. GALR2 antagonist inhibited anorexic leptin action and leptin-induced decreases in body weight gain, whereas GALR3 antagonist failed to induce a significant effect on leptin action (Figure 5A,B). Next, we determined the effect of GALR2 and GALR3 antagonists on SPX-induced changes in POMC promoter-luciferase activity in mouse hypothalamic neuronal mHypoA cells (Figure 5C). The SPX-induced increase in POMC promoter activity was inhibited by GALR2 antagonist, M871. However, GALR3 antagonist did not significantly affect SPX-induced POMC promoter activity. Together, these findings indicate that GALR2 is involved in SPX’s effects on leptin-induced anorexia.

## 4. Discussion

While previous studies have reported SPX as a possible satiety factor for the regulation of energy metabolism in multiple experimental models [4,15,16,40], detailed underlying mechanisms for SPX action in metabolism regulation have not yet been clearly understood. In this study, we found that central SPX induces anorexia and increases hypothalamic POMC mRNA expression. Additionally, we identified effect of SPX on leptin’s action in the control of food intake using a mouse model. Therefore, our results suggest a contribution of SPX to the leptin-dependent and POMC-mediated metabolism regulation within the brain.

A primary focus of this study was to elucidate a possible SPX action in the leptin-induced regulation of energy homeostasis. In the central nervous system, both SPX and leptin receptors are widely distributed throughout the entire brain structure [41,42,43]. However, for the central metabolism regulation, the hypothalamus has been well characterized for the leptin-dependent appetite behavior and energy balance [44,45,46,47,48]. Therefore, we tried to determine the hypothalamic site(s) where interaction between SPX and leptin occurs. Interestingly, icv administration of leptin increased SPX mRNA level in the ARC (Figure 2B), but not in other hypothalamic nuclei such as PVN, LH and SCN, suggesting that the ARC might be a key site for leptin-SPX signaling in the metabolism regulation. Indeed, the ARC is the main site for control of energy balance; it is located near the third ventricle and the median eminence that facilitates accessibility of circulating hormones and nutrients. In particular, the ARC is home to the orexigenic NPY/AgRP and anorexigenic POMC neurons that respond to circulating leptin [18,19,20,29]. In line with this, we observed that SPX is expressed in ARC cells expressing leptin receptor ObRb. Moreover, analysis using a Ribo-Tag system revealed that leptin induced a significant increase in translating mRNA species of SPX in ObRb-positive hypothalamic cells. These results suggest a possible involvement of hypothalamic SPX in the mediation of anorexigenic leptin action. To verify this hypothesis, we used two different approaches: (1) in vivo loss-of-function testing of hypothalamic SPX by icv administration of AS ODN, which resulted in inhibition of leptin-induced physiological and molecular changes, i.e., decreases in food intake and body weight gain and a concurrent increase in hypothalamic POMC mRNA expression; (2) icv administration of GALR2 antagonist, which also inhibited leptin-induced physiological and molecular changes. These results together indicate that SPX is, at least in part, involved in leptin-induced changes in food intake and POMC gene expression via GALR2 signaling in the hypothalamic ARC.

In addition to the ARC POMC neurons, multiple investigations have also recognized metabolic effects of leptin receptors in other brain regions and/or cell types. Within the ARC, leptin receptors are also expressed in glial cells and GABAergic local inhibitory cells where leptin receptors are suggested to play a role in the metabolism regulation through modification of the intra-ARC synaptic networks [49,50,51,52]. On the other hand, leptin receptors are present in multiple brain regions adjacent to the ARC, such as the ventromedial hypothalamic nucleus (VMH), LH, and dorsomedial hypothalamic nucleus (DMH), and the leptin receptor-expressing cells in these regions actively participate in the leptin-mediated appetite behavior and energy homeostasis [44,45,46]. Importantly, administration of leptin into the fourth ventricle induced phosphorylation of STAT3 in the ARC as well as the VMH and DMH, and inhibited food intake and body weight gain [53,54,55]. In line with these results, leptin is able to modulate midbrain dopaminergic cellular activity directly or indirectly and modulates feeding behavior through the dopaminergic system [46,56,57]. These results together support a concept that subsequent coordination of multiple brain regions is necessary for leptin-dependent weight loss. Collectively, although we paid our attention to the ARC POMC cells in this study, these previous reports suggest a possible involvement of other mechanisms, by a brain region- and cell type-dependent manner, in the leptin-dependent and SPX-mediated energy homeostasis. Further investigations are necessary to address these points of view.

In this study, we report that GALR2 is involved in SPX’s effects on leptin-induced anorexia and POMC expression. However, previous studies reported that the effect of SPX on the food intake is mediated by GALR3, based on the observation that the GALR3 antagonist SNAP inhibited SPX’s effects on cumulative food intake in mice [16,40]. It is unclear what caused this difference. The discrepancy may be due to different treatment methods such as intraperitoneal (ip) vs. icv injection of the antagonists and/or due to relatively short-term (1 h, 6 h) vs. long-term (24 h) observations of food intake and body weight gain between these studies (ip and 1 and 6 h) and ours (icv and 24 h). Moreover, they reported GALR3′s effect on the SPX action on food intake and body weight gain without leptin treatment, while we examined the effects of GALR2 and GALR3 antagonists on leptin-induced changes in physiology. Interestingly, a degree of the metabolic effects with GALR2 antagonist in the leptin-induced food intake and body weight gain is somewhat weaker than that from the AS ODN-dependent blockade of SPX biosynthesis (Figure 3B,C vs. Figure 5A,B). This discrepancy presumably suggests a possible existence of other unknown SPX receptor(s) in addition to GALR2. However, it could also be possible that the efficiency of AS ODN could be higher than that of the pharmacological antagonist in our experimental sets. In any case, these results indicate that leptin-induced food intake and body weight gain are, at least in part, SPX- and GALR2-dependent.

Although peripheral SPX injection resulted in an increase in locomotor activity in a previous study [11], our study did not find any change in body temperature or locomotor activity. This discrepancy might be due to different experimental settings such as ip vs. icv injection of SPX and the use of a diet-induced obese mice model vs. normal mice, respectively, in the previous study and in this study. Different SPX actions on energy intake and energy expenditure might also be explained by the differential effect of SPX on different neuronal populations responsible for energy consumption and energy intake. For example, POMC neurons consist of distinct heterogeneous populations that are differentially affected by leptin, serotonin and insulin and thus regulate energy metabolism via distinct melanocortin pathways [58]. Our histochemical results showed that only 70% of ObRb-expressing cells also express SPX, suggesting that there are different subpopulations of SPX-expressing cells and that leptin affects SPX expression only through the subpopulation of SPX cells that express ObRb. Thus, SPX cells without ObRb expression may be involved in pathways other than mediation of leptin action.

Finally, the results from the present study have not clarified why a part of leptin’s action requires mediatory SPX function in the regulation of target molecules and physiology. While this will require further studies of the underling mechanisms, central SPX function might contribute to elaborate consolidation of subtle distinctions of many diverse peripheral inputs.

In summary, the present findings identify SPX as a novel component involved in leptin-mediated regulation of energy homeostasis.

## 5. Conclusions

**Figure 6:**
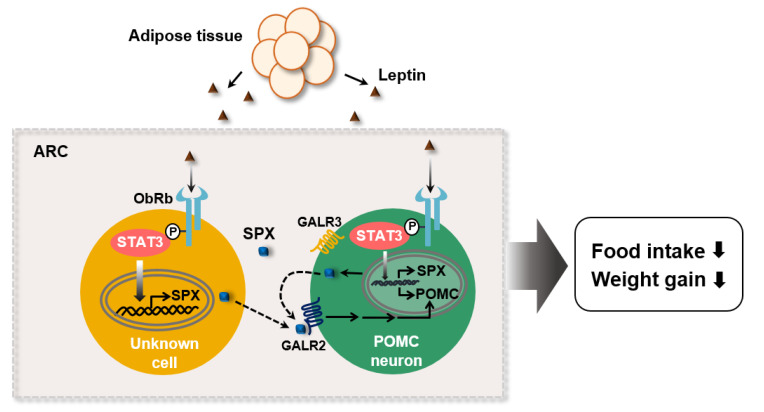
**Proposed model for SPX action in leptin-mediated control of food intake within the hypothalamic ARC.** SPX is synthesized under a control of leptin in the ObRb expressing cells and acts on POMC expression via GALR2 receptor signaling. Dashed lines represent autocrine and/or paracrine SPX action.

In this study, we demonstrated that SPX is involved in the leptin’s action for feeding behavior via regulation of POMC transcription (Figure 6). Leptin promotes SPX transcription through STAT3 activation in the ObRb expressing cells of the hypothalamic ARC. In turn, SPX increases POMC gene expression via GALR2 signaling, resulting in a decrease in food intake and body weight gain. As of now, because it is unclear which cell expresses SPX (e.g., POMC, AgRP, GABAergic neuron, etc.) in the ARC, we suggest a possible SPX function as an autocrine factor by POMC neuron or a paracrine factor by neighboring cells expressing ObRb.

## Data Availability

Data are contained within the article.

## Supplementary Materials

The following supporting information can be downloaded at: https://www.mdpi.com/article/10.3390/biom12020236/s1, Supplementary Figure S1: Effect of leptin on SPX expression in the hypothalamic nuclei; Supplementary Figure S2: Double immunofluorescence image showing SPX expression in ObRb-positive cells; Supplementary Figure S3: The Ribo-Tag system of transgenic (*ObRb-Cre;Rpl22^HA^*) mice.
