# Supplementary material for: Spexin Regulates Hypothalamic Leptin Action on Feeding Behavior
Source: Biomolecules. 2022 Jan 31;12(2):236. doi: 10.3390/biom12020236 (PMC8961618; doi:10.3390/biom12020236)
Supplement: Supplementary file 1 [file biomolecules-12-00236-s001.zip › biomolecules-1535184-supplementary.pdf]

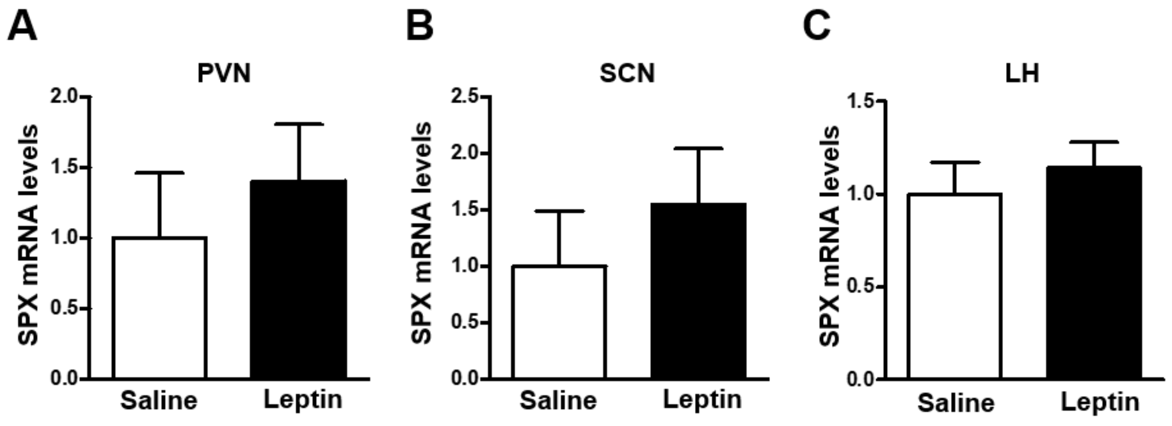

Supplementary Figure S1. Effect of leptin on SPX expression in the hypothalamic nuclei. To determine the effect of leptin on SPX expression in the hypothalamic nuclei, eight-week-old male mice were fasted for 24 h and icv-injected with leptin (2.5 μg). Areas were micropunched out 1 h after leptin injection. Total RNA was isolated from the paraventricular nucleus (PVN) (A), suprachiasmatic nucleus (SCN) (B) and lateral hypothalamus (LH) (C) and analyzed with qRT-PCR.

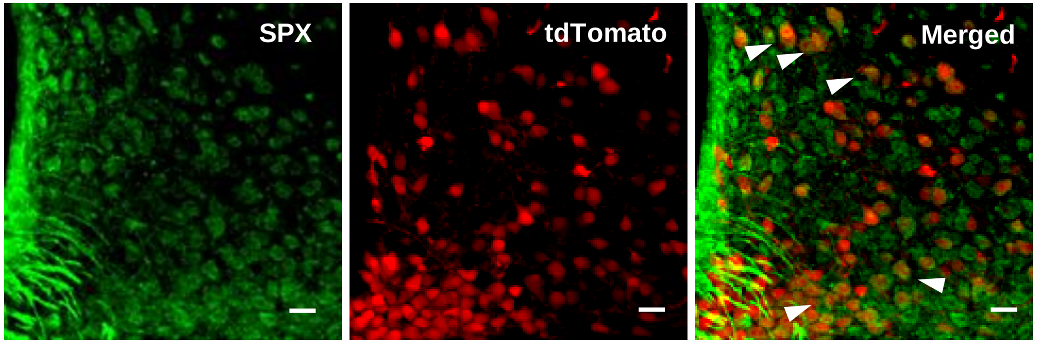

Supplementary Figure S2. Double immunofluorescence image showing SPX expression in ObRb-positive cells. To identify SPX expression in cells expressing leptin receptor ObRb, we performed immunohistochemical analysis of SPX (green) in the tdTomato reporter-expressing cells (red) of ObRb-Cre;Ai14 mice. Arrowheads indicate representative SPX expression in ObRb-positive cells. Scale bar = 20 μm.

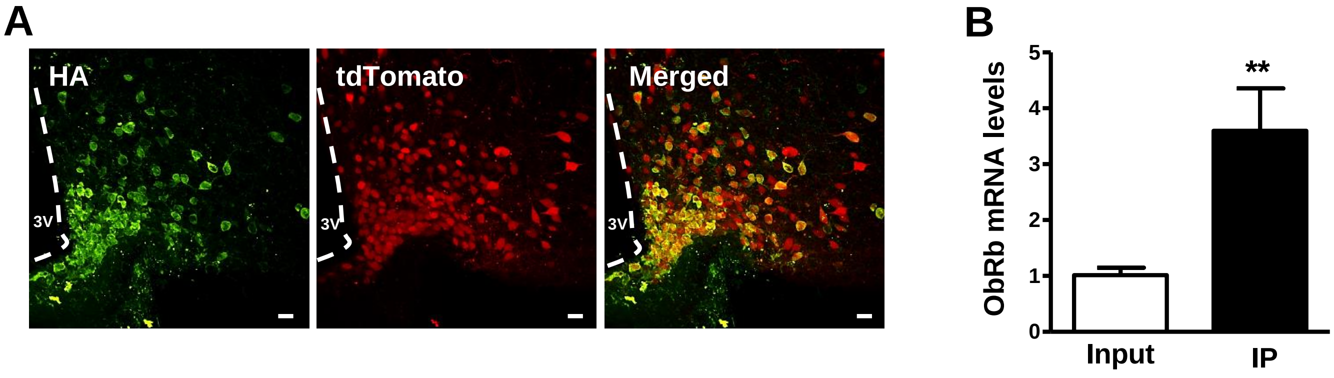

Supplementary Figure S3. The Ribo-Tag system of transgenic (*ObRb-Cre;Rpl22^{HA}*) mice. (A) Immunohistochemical analysis was performed on coronal brain sections from ObRb-Cre;*Rpl22*^{HA};Ai14 mice and showed that most ObRb-positive cells visualized with tdTomato reporter expression (red) were immuno-stained with anti-HA staining (green). Scale bar = 20 μm. (B) MBH extracts from ObRb-Cre;*Rpl22*^{HA} mice were immunoprecipitated (IP) with anti-HA antibody. The IP and input RNA were analyzed using qRT-PCR analysis with a PCR primer set specific to ObRb mRNA. Input represents the samples extracted from the mouse MBH before immunoprecipitation. **, P<0.01.
